# Prognosis and Nomogram Prediction for Patients with Oral Squamous Cell Carcinoma: A Cohort Study

**DOI:** 10.3390/diagnostics13101768

**Published:** 2023-05-17

**Authors:** Xin-Yuan Zhang, Shang Xie, Dian-Can Wang, Xiao-Feng Shan, Zhi-Gang Cai

**Affiliations:** Department of Oral and Maxillofacial Surgery, Peking University School and Hospital of Stomatology & National Center of Stomatology & National Clinical Research Center for Oral Diseases & National Engineering Research Center of Oral Biomaterials and Digital Medical Devices, Beijing 100081, China

**Keywords:** oral squamous cell carcinoma, nomogram, prognosis

## Abstract

The TNM staging system is often used to predict the prognosis of patients with oral squamous cell carcinoma (OSCC). However, we have found that patients under the same TNM staging may exhibit tremendous differences in survival rates. Therefore, we aimed to investigate the prognosis of postoperative OSCC patients, establish a nomogram survival prediction model, and verify its effectiveness. Operative logs were reviewed for patients who underwent surgical treatment for OSCC at the Peking University School and Hospital of Stomatology. Patient demographic and surgical records were obtained, and they were followed up for overall survival (OS). A total of 432 patients with oral squamous cell carcinoma were included in the study, with a median follow-up time of 47 months. Based on the results of the Cox regression analysis, we constructed and verified the nomogram prediction model, which includes gender, BMI, OPMDs, pain score, SCC grade, and N stage. The C-index value of the 3-year and 5-year prediction models was 0.782 and 0.770, respectively, proving that the model has a certain level of prediction stability. The new nomogram prediction model has potential clinical significance for predicting the postoperative survival of OSCC patients.

## 1. Introduction

The oral cavity plays an important role in physiological functions, such as appearance, speech, chewing, and swallowing. Its negative impact on the psychology of patients and their families cannot be overlooked [1]. Oral squamous cell carcinoma (OSCC) is one of the most common malignant tumors in the head and neck, with approximately 52,000 new cases reported each year in China [2,3]. Despite the increasing maturity of surgical radical treatment and reconstruction technologies for OSCC, studies have shown that the 5-year survival rate has not significantly improved over the past 20 years [4,5,6,7]. In our clinical practice, we have observed that most patients with OSCC who undergo surgery in our hospital have a better prognosis compared to previous reports. Therefore, we investigated to verify our clinical findings.

The TNM staging system is commonly used to predict the prognosis of OSCC and guide doctors in choosing an appropriate treatment plan in clinical practice. However, we found that the survival of patients under the same TNM staging can vary significantly. This suggests that the TNM staging system has certain limitations, mainly because it only focuses on the characteristics of the disease itself while ignoring the influence of other prognostic factors, such as patient sociodemographic factors, personal circumstances, and psychosocial factors. A recent study has shown that even though the American Joint Committee on Cancer (AJCC) included the depth of tumor invasion and lymph node extracapsular metastasis factors into the TNM staging in the eighth edition of the staging manual, personalized prognostic judgment is still necessary [8,9]. Therefore, it is essential to study the prognostic factors of OSCC further and construct a prognostic prediction model suitable for individuals [10].

As a historical mathematical model, the nomogram was first proposed by a French engineer in 1884 for engineering application. It can quickly, intuitively, and accurately explain the relationship between different variables graphically through complex engineering mechanical formulas [11]. In recent years, the nomogram has been increasingly used in the medical field and is now a widely used prediction model in clinical research. It can take complex regression equations, such as logistic regression and Cox regression, and display them visually through various influencing factors on outcome variables to assign high and low point levels. The scores corresponding to various factors affecting the outcome can be combined. The total score and outcome probability function conversion relations between incidents can be used to quickly calculate the probability and survival rate of specific events and other indicators [11,12,13].

Currently, nomograms are also widely used to assess the prognosis of a wide range of tumors, providing reliable prognostic information tailored to each patient to assist doctors in developing treatment plans, including lung cancer [14,15], colorectal cancer [16,17,18], liver cancer [19], breast cancer [20], and so on. However, most nomograms for head and neck tumors were established based on the Surveillance, Epidemiology, and End Results (SEER) database. Therefore, these models may not apply to the Chinese population. The aim of this study was to develop and validate a nomogram using Chinese data that can accurately predict overall survival in individual patients based on available information on patients with OSCC.

## 2. Materials and Methods

### 2.1. Patients

A retrospective analysis was performed on patients with primary OSCC who underwent surgery at the Second and Fourth wards of the Department of Oral and Maxillofacial Surgery at Peking University Hospital of Stomatology between May 2014 and May 2017.

### 2.2. Inclusion Criteria

The inclusion criteria were: (a) Patients with postoperative pathological diagnosis of OSCC; (b) Patients with complete clinical records.

### 2.3. Exclusion Criteria

Patients with poor compliance and loss of follow-up were excluded.

### 2.4. Data Collection

The following patient and tumor characteristics were collected: age, gender, education level, body mass index (BMI), anxiety state score (using the anxiety thermometer created by Roth [21], 0–2 points without obvious anxiety; 3–5 points of mild anxiety; 6–8 points of moderate anxiety; 9–10 points of severe anxiety), smoking history, drinking history, family history, overall comorbidity grade (assessed by adult comorbidity; 27 items on the scale [22], 0 is none, 1 is mild, 2 is moderate, and 3 is severe), oral potentially malignant disorders (OPMDs) history, the subsite of the tumor within the oral cavity, pain severity score (score using a digital pain scale, 0–2 points for no obvious pain; 3–5 points for mild pain; 6–8 points for moderate pain; 9–10 points for severe pain), TNM stage (according to the AJCC 7th edition of the staging system), and pathologic grade.

The study endpoint was overall survival (OS), which was calculated from the date of surgery to the date of death or the last follow-up visit.

### 2.5. Nomogram Construction and Validation

Statistical analysis was performed using SPSS 25.0 (IBM, Armonk, NY, USA) software. The chi-square test was used for categorical variables to compare differences between groups. Univariate and multivariate Cox proportional hazards regression models were used to estimate the predictive effect of each variable on patient survival. A nomogram was formulated based on the results of the multivariate analyses.

Further nomogram construction and validation were performed with R software 4.0.1 and the Survival, RMS, Hmisc, and Foreign packages. Harrell concordance Index (C-index) values were calculated using 1000 bootstrap resampling for discrimination testing. The consistency of the model was evaluated by drawing a calibration curve. Finally, according to the predicted results, the research patients were divided into high, medium, and low-risk groups, and a Kaplan-Meier survival analysis and Log-rank test of survival data were performed. When *p* < 0.05, the difference was statistically significant.

## 3. Results

### 3.1. Patient Characteristics and Risk Factors of OSCC

After initial screening, 509 patients with complete clinical records were followed up. A total of 77 patients were lost to follow-up, leading to a loss rate of 15.13%. Finally, 432 patients were included as research patients, with a median follow-up time of 47 months. Table 1 summarizes demographic information and the clinicopathologic features of the 432 patients. Among these patients, 112 patients had tumor recurrence. The recurrence rate was 25.93% (112/432). A total of 85 patients were diseased, with a mortality rate of 19.68% (85/432). The overall survival at three and five years after surgery were 84.43% and 77.64%, and the recurrence-free survival at three and five years were 77.29% and 71.89%, respectively.

Then, a Chi-Square test was conducted between the surviving and deceased groups. In the deceased group, the mortality rate of male patients was significantly higher than that of female patients (*p* = 0.006). BMI < 18.5 group had the highest mortality, and the difference was statistically significant (*p* = 0.008). Smoking group mortality was significantly higher than the non-smoking group (*p* = 0.021). The mortality of the drinking group was higher than that of the non-drinking group (*p* = 0.008). Among the patients in the deceased group, 4/64 (6.25%) in the OPMDs group and 81/368 (22.01%) in the non-OPMDs group had higher mortality, which may be due to the small number of patients in the OPMDs group and the presence of close follow-up after precancerous lesions (*p* = 0.003). In the groups of pain caused by the primary tumor, the higher the pain scores, the higher the mortality rate, and the difference was statistically significant (*p* < 0.0001). The mortality rate increased gradually for the T and N stages of SCC with significant differences (*p* < 0.0001). Detailed data are shown in Table 2.

A total of nine risk factors were identified with statistically significant contributors (*p* < 0.05). Gender, BMI, smoking history, drinking history, OPMDs, pain scores, SCC grade, T stage, and N stage were further included in a univariate Cox regression analysis model for further analysis, and detailed data are shown in Table 3. In gender groups, the Hazard Ratio (HR) of male patients was set to 1, and that of female patients was 0.487, *p* < 0.05, indicating a statistically significant difference. In the BMI group, the risk was set as I in the group less than 18.5, and the HR value was 0.767 in the 18.5–24 group and 0.481 in the group greater than 24. The difference was statistically significant in the group greater than 24 (*p* < 0.05). In the smoking group, the risk of the non-smoking group was set as 1, and the HR value of the smoking group was 1.632; the difference was statistically significant (*p* < 0.05). In the drinking group, the risk of the non-drinking group was set as 1, and the HR value of the drinking group was 1.735, and the difference was statistically significant (*p* < 0.05). In the group of OPMDs, the risk of the group without OPMDs was set as 1, and the HR value of the group with OPMDs was 0.279, with a statistically significant difference (*p* < 0.05). In the grouping of pain degree, the risk of the 0–2 group was set as 1, the HR of the 3–5 group was 1.786, the 6–8 group was 3.223, and the 9–10 group was 6.847; the difference was statistically significant (*p* < 0.05). In SCC grade, the risk of grade 1 was set as the HR value of grades 1 and 2 was 2.009, that of grade 3 was 4.635, and that of grade 4 was 4.558, and the difference was statistically significant (*p* < 0.05). In the T stage, the risk of T1 was set as the HR value of 1, the HR value of T2 was 2.047, T3 was 3.286, and T4 was 3.654; the difference was statistically significant (*p* < 0.05). In the N stage, the risk of N0 was set as 1, and the HR value of N1 was 2.711, N2b was 3.040, N2c was 8.624, and N3 was 47.613, with statistically significant differences (*p* < 0.05). Detailed data are shown in Table 3.

After univariate Cox regression analysis, the risk factors with statistically significant differences (*p* < 0.05) were as follows: gender, BMI, smoking history, drinking history, OPMDs, pain score, SCC grade, T stage, and N stage were further analyzed by multivariate Cox regression analysis model. The results showed that BMI (≥24), pain score (6–8, 9–10), SCC grade, and N stage were independent risk factors for mortality. Detailed data are shown in Table 3.

### 3.2. Nomogram Construction and Validation

**Figure 1:**
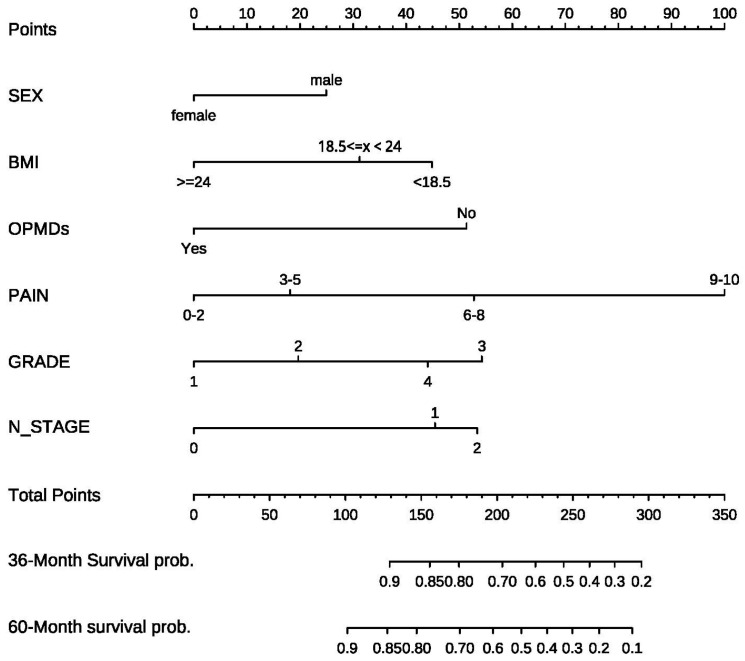
Nomogram for predicting 3- and 5-year overall survival (OS) in oral squamous cell carcinoma (OSCC) patients with surgical treatment.

In combination with the above univariate and multivariate Cox regression results, it was necessary to screen the final variables included in the model to establish an effective column chart. Stepwise regression was used to select the optimal model. Finally, six factors, including gender, BMI, OPMDs, pain score, SCC grade, and N stage, were included. Variables were assigned and line charts were drawn. As shown in Figure 1, the first line of the column chart represents the integral line, and the second to seventh lines represent the possible values of the six variables. It is important to emphasize that there was only one patient with the N3 stage among 432 patients; according to the requirements of the model, they were divided into three groups, 0 representing the N0 stage, 1 representing the N1 stage, and 2 representing the N2 and N3 stages. According to the value of different variables, the corresponding integral can be found on the integral line of the first row of the rosette line by vertical line, and the integral value ranges from 0 to 100 points. Then, the total score can be obtained by adding the scores of each risk factor. Below the column line, the ratio of 3-year and 5-year survival probability is obtained by the vertical line according to the position of the total score line.

The C-index is used to judge the differentiation degree of the model to evaluate the prediction efficiency of the model. The value of C-index is between 0.5–1. 0.5 means completely inconsistent, indicating that the model has no prediction effect, and 1 means completely consistent, indicating that the prediction result of the model is completely consistent with reality. It is generally considered that a C-index between 0.50 and 0.70 is of low accuracy, 0.71–0.90 is of medium accuracy, and higher than 0.90 is of high accuracy. The bootstrap self-sampling method was adopted in this study, and the C-index was calculated by repeated sampling 1000 times. The C-index value of the 3-year prediction model and 5-year prediction model in this study was 0.782 and 0.770, indicating that the model had medium accuracy and good differentiation.

**Figure 2:**
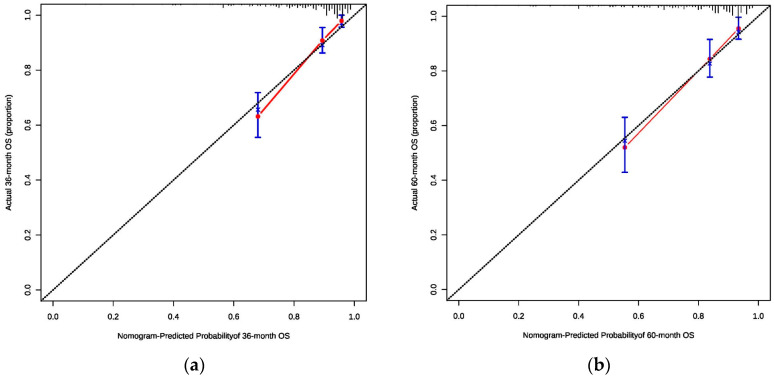
(**a**) The consistency calibration curves of the nomogram for oral squamous cell carcinoma (3-year); (**b**) The consistency calibration curves of the nomogram for oral squamous cell carcinoma (5-year).

Consistency is an indicator that reflects the degree of coincidence between the predicted probability and the actual probability. The consistency calibration curve is a curve that predicts the survival and the actual survival through fitting after Cox regression with the score of the line graph as the influencing factor. The abscissa is the survival probability predicted by the line graph, and the ordinate is the actual survival observed. The standard curve is a line that passes through the origin of coordinates, and the line of data points fits with the diagonal line approximately, indicating that the model has better calibration ability, as shown in Figure 2a,b. The consistency of this study is ideal.

**Figure 3:**
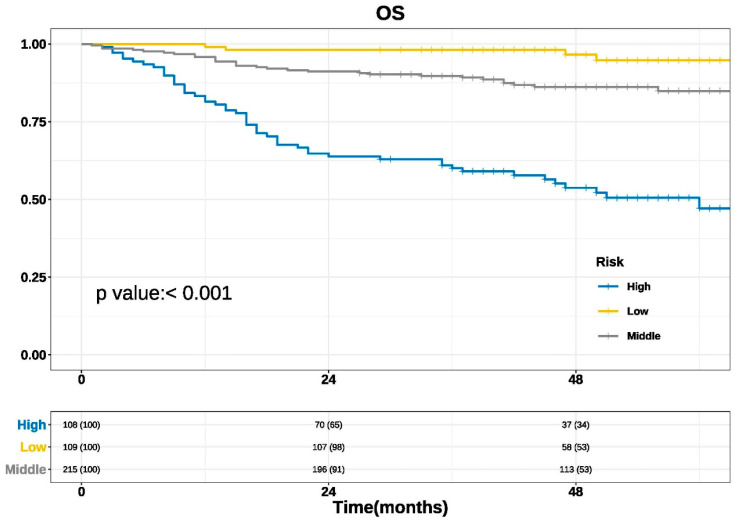
Kaplan-Meier survival curves based on cut-off value using the total predicted scores assigned by the nomogram.

Using our established nomogram, the total predicted scores of 432 patients in the study were arranged from small to large, and the study patients were grouped by quartile. The first quartile Q1 and the third quartile Q3 were used as cut-off values, and the study patients were divided into low-risk, medium-risk, and high-risk groups. Kaplan-Meier survival analysis and a log-rank test were performed for survival data. The results are shown in Figure 3 (*p* < 0.001), indicating a statistically significant difference indicating good clinical practicability of the nomogram.

## 4. Discussion

With the advancement of surgical techniques and reconstruction, complete tumor resection has achieved favorable results. Thus, the patients of OSCC have a better prognosis than they did 20 years ago, verified by our statistics [23,24]. Here we developed a new model based on the data of this research center that can accurately predict cancer survival in patients with OSCC.

In this study, the prognostic prediction model of the 3-year and 5-year survival probability of OSCC was established. Six factors, including gender, BMI, OPMDs, pain score, SCC grade, and N stage, were included based on Cox regression analysis. For the first time, the pain score and BMI value of the tumor source were included in the prediction model. Through internal verification, the C-index of the 3-year and 5-year prediction models were 0.782 and 0.770, respectively, which proves that it has good forecasting ability. Through the more accurate curve, the survival condition and the actual survival condition were fitted, indicating that the calibration ability and the consistency of the model were ideal. Through the survival analysis of patients with high, middle, and low risk, the prediction model proved to be more practical in the clinic.

Until now, 7 studies focused on oral cancer nomograms, accounting for 16% of the 44 studies of head and neck tumor nomograms [25]. Among them, one review focused on OSCC nomograms [26], two studies focused on OSCC [27,28], one study focused on patients with buccal squamous cell cancer [29], and three studies (reported in 2017, 2018, and 2019, respectively) were for patients with tongue squamous cell cancer [30,31,32]. Montero P. et al. [28] reviewed 1617 patients with oral squamous cell carcinoma from 1985 to 2009 in a cancer center in the United States and established a nomogram to predict overall survival, tumor-specific survival, and local recurrence probability. The main variables included in overall survival were age, race, smoking status, tumor size, and N stage. The internal validation C index was 0.67. The main factors included in predicting tumor-specific survival and recurrence probability were tumor size, lymph node metastasis, lesion site, and presence of bone tissue invasion. The C-indices were 0.66 and 0.60, different from 0.782 and 0.770, internally verified in the prediction of 3-year and 5-year survival in this study. However, the data of this study is from 1985 and 2009. In 2016, Bobdey S. et al. [27] reviewed 609 patients with oral squamous cell carcinomas in an Indian hospital from 2006 to 2008. Established criteria included age, presence of systemic conditions, disease stage, with or without lymph node metastasis, tumor thickness, presence of nerve infiltrating nomogram to predict overall survival, different thicknesses in the tumors, and the tumor infiltration depth, according to the study of tumor thickness 0.8 cm group. The verification C-index was 0.72, but the study lacked the extraction of patients’ social psychology and other influencing factors. The classification of some influencing factors was relatively simple, such as systemic conditions and lymph node metastasis. In 2018, Bobdey S. et al. [29] reviewed 205 patients with T4 buccal squamous cell carcinoma who underwent surgical treatment from 2009 to 2014 were reviewed. An overall survival curve was established, including tumor differentiation, lymph node metastasis, presence or absence of nerve invasion, and presence or absence of bone destruction, with a C-index of 0.7266. However, all the patients included in this study were patients with T4 buccal squamous cell carcinoma undergoing surgical treatment in this medical center in India, and some patients with advanced buccal squamous cell carcinoma that could not be treated surgically were ignored, leading to a certain selection bias in this model. In 2017, Li Y. et al. [30] used The Surveillance Epidemiology and End Results (SEER) database to review 7587 surgical patients and 5087 nonsurgical patients diagnosed with tongue squamous cell carcinoma from 2004 to 2013. The main variables included age, race, marital status, TNM stage, and grade. Due to the large sample size, the TNM stage was more detailed than the rosette of this study, and the C-indexes of internal verification were all greater than 0.7. In this study,191 patients with tongue squamous cell carcinoma from a public hospital in China were used for external verification of the surgical group’s nomograms. The C-index was 0.691, indicating that the mode had a certain predictive ability. However, its calibration curve is not ideal. At the same time, according to the data obtained from the SEER database, some important factors affecting prognosis are missing. Additionally, influential factors, such as race and marital status, do not apply to domestic predictions. In 2018, Chang B. et al. [32] reviewed 235 patients with OSCC with R0 resection initially treated with surgery in a cancer center in Southern China from 2000 to 2007 and established a nomogram. Meanwhile, another prospective cohort initially treated with surgery from 2008 to 2012 was used as a validation cohort. Include age, N stage, tumor infiltration depth, and range were cleaning the 5-year survival rate of prediction, the internal validation and external validation index exceeded 0.7, predict performance better, the study for the first time the tumor infiltration depth into the nomogram, compared to this study, for predicting the prognosis of tongue squamous carcinoma may be an advantage, but it did not take into account factors such as pain and BMI. In 2019, Sun W. et al. [31] enrolled 1550 patients with stage IV tongue squamous cell carcinoma in the SEER database, and the training set and validation set were established according to 7:3. Finally, the nomogram including age, race, marital status, site of onset, and TNM stage was established. The C-index of internal validation was 0.708, and the C-index of external validation was 0.664. This study was also derived from the SEER database and had similar limitations as the above study.

However, our research also has several limitations. Firstly, as this study did not record the tumor infiltration depth, degree of blood-nerve invasion, and lymph node invasion, further research needs to be added. Secondly, this study belongs to the single-center study; the scope of application is limited and still needs multi-center. Thirdly, the sample size is not large. Thus, in the future, large samples and long-term follow-up will be needed to improve the accuracy of the prediction model and expand its scope of application.

## 5. Conclusions

From a clinical perspective, we found that advancing surgical approaches and reconstruction offer a better prognosis for patients with OSCC. The new nomogram prediction model has potential clinical significance in predicting postoperative survival of OSCC, which can help clinicians during consultation, treatment planning, follow-up, and postoperative risk assessment.

## Figures and Tables

**Table 1:** Basic personal information of the patients.

Variable	No. of Patients (%)	
Age		
<30	6	1.39%
30 ≤ x < 40	26	6.02%
40 ≤ x < 50	63	14.58%
50 ≤ x < 60	124	28.71%
60 ≤ x < 70	140	32.41%
70 ≤ x < 80	63	14.58%
≥80	10	2.31%
Gender		
Male	291	67.36%
Female	141	32.64%
Educational level		
Higher	114	26.39%
Medium	108	25.00%
Lower	210	48.61%
BMI		
<18.5	25	5.79%
18.5 ≤ x < 24	208	48.15%
≥24	199	46.06%
Anxiety state score		
0–2	216	50.00%
3–5	127	29.40%
6–8	78	18.05%
9–10	11	2.55%
Smoking		
Yes	226	52.31%
No	206	47.69%
Drinking		
Yes	169	39.12%
No	263	60.88%
Tumor history		
Yes	45	10.42%
No	387	89.58%
Family history		
Yes	156	36.11%
No	276	63.89%
Overall comorbidity grade		
0	216	50.00%
1	173	40.05%
2	33	7.64%
3	10	2.31%
OPMDs history		
Yes	64	14.81%
No	368	85.19%
Subsite		
Lip	19	4.40%
Buccal mucosa	43	9.95%
Gingiva	94	21.76%
Retromolar tissue	15	3.47%
Palate	7	1.62%
Mouth floor	52	12.04%
Tongue	179	41.44%
Oropharyngeal	23	5.32%
Pain severity score		
0–2	191	44.22%
3–5	149	34.49%
6–8	70	16.20%
9–10	22	5.09%
SCC grade		
1	181	41.90%
2	154	35.65%
3	76	17.59%
4	21	4.86%
T stage		
1	136	31.48%
2	161	37.27%
3	49	11.34%
4	86	19.91%
N stage		
0	285	65.98%
1	63	14.58%
2b	70	16.20%
2c	13	3.01%
3	1	0.23%
Radiation and chemotherapy		
Yes	118	27.31%
No	314	72.69%

**Table 2:** The Chi-Square test between the survived and deceased groups.

Variable	The Deceased Groups (*n* = 85, 19.68%)		The Survived Groups (*n* = 347, 80.32%)		*p*
Gender					
Male	68	23.37%	223	76.63%	0.006
Female	17	12.06%	124	87.94%	
BMI					
<18.5	8	32.00%	17	68.00%	0.008
18.5 ≤ x < 24	50	24.04%	158	75.96%	
≥24	27	13.57%	172	86.43%	
Smoking					
Yes	54	23.89%	172	76.11%	0.021
No	31	15.05%	175	84.95%	
Drinking					
Yes	44	26.04%	125	73.96%	0.008
No	41	15.59%	222	84.41%	
OPMDs					
Yes	4	6.25%	60	93.75%	0.003
No	81	22.01%	287	77.99%	
Pain scores					
0–2	22	11.52%	169	88.48%	<0.0001
3–5	30	20.13%	119	79.87%	
6–8	22	31.43%	48	68.57%	
9–10	11	50.00%	11	50.00%	
SCC grade					
1	18	9.94%	163	90.06%	<0.0001
2	29	18.83%	125	81.17%	
3	39	45.88%	46	54.12%	
4	8	38.10%	13	61.90%	
T stage					
1	13	9.56%	123	90.44%	<0.0001
2	31	19.25%	130	80.75%	
3	14	28.57%	35	71.43%	
4	27	31.40%	59	68.60%	
N stage					
0	34	11.93%	251	88.07%	<0.0001
1	18	28.57%	45	71.43%	
2b	23	32.86%	47	67.14%	
2c	9	69.23%	4	30.77%	
3	1	100.00%	0	0.00%	

**Table 3:** Significance of clinicopathologic features by Cox regression analysis.

Variable	Univariate		Multivariate	
	Hazard Ratio (95% CI)	*p*	Hazard Ratio (95% CI)	*p*
Gender				
Male	1		1	
Female	0.487 (0.286–0.828)	0.008	0.561 (0.280–1.125)	0.103
BMI				
<18.5	1		1	
18.5 ≤ x < 24	0.767 (0.364–1.618)	0.486	0.756 (0.352–1.623)	0.472
≥24	0.418 (0.190–0.921)	0.03	0.434 (0.192–0.978)	0.044
Smoking				
No	1		1	
Yes	1.632 (1.049–2.538)	0.03	0.771 (0.396–1.502)	0.444
Drinking				
No	1		1	
Yes	1.735 (1.134–2.656)	0.011	0.949 (0.529–1.703)	0.861
OPMDs				
No	1		1	
Yes	0.279 (0.102–0.761)	0.013	0.413 (0.145–1.173)	0.097
Pain				
0–2	1		1	
3–5	1.786 (1.030–3.097)	0.039	1.383 (0.771–2.479)	0.277
6–8	3.223 (1.783–5.826)	<0.0001	2.301 (1.222–4.334)	0.01
9–10	6.847 (3.307–14.177)	<0.0001	5.193 (2.269–11.885)	<0.0001
Grade				
1	1		1	
2	2.009 (1.116–3.618)	0.02	1.352 (0.735–2.485)	0.332
3	4.635 (2.583–8.316)	<0.0001	2.393 (1.264–4.528)	0.007
4	4.558 (1.980–10.491)	<0.0001	2.129 (0.842–5.386)	0.111
T stage				
1	1		1	
2	2.047 (1.071–3.913)	0.03	1.508 (0.764–2.979)	0.237
3	3.286 (1.544–6.993)	0.002	1.864 (0.833–4.174)	0.13
4	3.654 (1.885–7.083)	<0.0001	1.184 (0.560–2.502)	0.659
N stage				
0	1		1	
1	2.711 (1.530–4.803)	0.001	2.164 (1.187–3.944)	0.012
2b	3.040 (1.790–5.162)	<0.0001	2.090 (1.158–3.771)	0.014
2c	8.624 (4.125–18.033)	<0.0001	3.134 (1.324–7.420)	0.009
3	47.613 (6.197–365.834)	<0.0001	14.848 (1.523–144.762)	0.02

## Data Availability

The data used to support the findings of this study are available from the corresponding author upon request.

## References

[1] Cramer J.D., Burtness B., Le Q.T., Ferris R.L. (2019). The changing therapeutic landscape of head and neck cancer. Nat. Rev. Clin. Oncol..

[2] Zhang S., Sun K., Zheng R., Zeng H., Wang S., Chen R., Wei W., He J. (2021). Cancer incidence and mortality in China, 2015. J. Natl. Cancer Cent..

[3] Zheng R., Zhang S., Zeng H., Wang S., Sun K., Chen R., Li L., Wei W., He J. (2022). Cancer incidence and mortality in China, 2016. J. Natl. Cancer Cent..

[4] Jemal A., Murray T., Ward E., Samuels A., Tiwari R.C., Ghafoor A., Feuer E.J., Thun M.J. (2005). Cancer statistics, 2005. CA Cancer J. Clin..

[5] Siegel R.L., Miller K.D., Fuchs H.E., Jemal A. (2021). Cancer Statistics, 2021. CA Cancer J. Clin..

[6] Siegel R.L., Miller K.D., Jemal A. (2015). Cancer statistics, 2015. CA Cancer J. Clin..

[7] Zeng H., Chen W., Zheng R., Zhang S., Ji J.S., Zou X., Xia C., Sun K., Yang Z., Li H. (2018). Changing cancer survival in China during 2003-15: A pooled analysis of 17 population-based cancer registries. Lancet Glob. Health.

[8] Moeckelmann N., Ebrahimi A., Tou Y.K., Gupta R., Low T.H., Ashford B., Ch’ng S., Palme C.E., Clark J.R. (2018). Prognostic implications of the 8th edition American Joint Committee on Cancer (AJCC) staging system in oral cavity squamous cell carcinoma. Oral Oncol..

[9] Kattan M.W., Hess K.R., Amin M.B., Lu Y., Moons K.G., Gershenwald J.E., Gimotty P.A., Guinney J.H., Halabi S., Lazar A.J. (2016). American Joint Committee on Cancer acceptance criteria for inclusion of risk models for individualized prognosis in the practice of precision medicine. CA Cancer J. Clin..

[10] Steyerberg E.W., Moons K.G., van der Windt D.A., Hayden J.A., Perel P., Schroter S., Riley R.D., Hemingway H., Altman D.G. (2013). Prognosis Research Strategy (PROGRESS) 3: Prognostic model research. PLoS Med..

[11] Grimes D.A. (2008). The nomogram epidemic: Resurgence of a medical relic. Ann. Intern. Med..

[12] Balachandran V.P., Gonen M., Smith J.J., DeMatteo R.P. (2015). Nomograms in oncology: More than meets the eye. Lancet Oncol..

[13] Weiser M.R., Gönen M., Chou J.F., Kattan M.W., Schrag D. (2011). Predicting survival after curative colectomy for cancer: Individualizing colon cancer staging. J. Clin. Oncol..

[14] Liang W., Zhang L., Jiang G., Wang Q., Liu L., Liu D., Wang Z., Zhu Z., Deng Q., Xiong X. (2015). Development and validation of a nomogram for predicting survival in patients with resected non-small-cell lung cancer. J. Clin. Oncol..

[15] Xie D., Marks R., Zhang M., Jiang G., Jatoi A., Garces Y.I., Mansfield A., Molina J., Yang P. (2015). Nomograms Predict Overall Survival for Patients with Small-Cell Lung Cancer Incorporating Pretreatment Peripheral Blood Markers. J. Thorac. Oncol..

[16] Weiser M.R., Landmann R.G., Kattan M.W., Gonen M., Shia J., Chou J., Paty P.B., Guillem J.G., Temple L.K., Schrag D. (2008). Individualized prediction of colon cancer recurrence using a nomogram. J. Clin. Oncol..

[17] Zhang J.X., Song W., Chen Z.H., Wei J.H., Liao Y.J., Lei J., Hu M., Chen G.Z., Liao B., Lu J. (2013). Prognostic and predictive value of a microRNA signature in stage II colon cancer: A microRNA expression analysis. Lancet Oncol..

[18] Zhou M., Lv S., Hou Y., Zhang R., Wang W., Yan Z., Li T., Gan W., Zeng Z., Zhang F. (2022). Characterization of sialylation-related long noncoding RNAs to develop a novel signature for predicting prognosis, immune landscape, and chemotherapy response in colorectal cancer. Front. Immunol..

[19] Ying H.Q., Deng Q.W., He B.S., Pan Y.Q., Wang F., Sun H.L., Chen J., Liu X., Wang S.K. (2014). The prognostic value of preoperative NLR, d-NLR, PLR and LMR for predicting clinical outcome in surgical colorectal cancer patients. Med. Oncol..

[20] Diao J.D., Ma L.X., Sun M.Y., Wu C.J., Wang L.J., Liu Y.L., Yang Y.J. (2019). Construction and validation of a nomogram to predict overall survival in patients with inflammatory breast cancer. Cancer Med..

[21] Roth A.J., Kornblith A.B., Batel-Copel L., Peabody E., Scher H.I., Holland J.C. (1998). Rapid screening for psychologic distress in men with prostate carcinoma: A pilot study. Cancer.

[22] Gao L., Mao C., Yu G.Y., Peng X. (2016). Authorization, translation, back translation and language modification of the simplified Chinese adult comorbidity-27 index. Zhonghua Kou Qiang Yi Xue Za Zhi.

[23] Greenlee R.T., Hill-Harmon M.B., Murray T., Thun M. (2001). Cancer statistics, 2001. CA Cancer J. Clin..

[24] Jemal A., Thomas A., Murray T., Thun M. (2002). Cancer statistics, 2002. CA Cancer J. Clin..

[25] Tham T., Machado R., Herman S.W., Kraus D., Costantino P., Roche A. (2019). Personalized prognostication in head and neck cancer: A systematic review of nomograms according to the AJCC precision medicine core (PMC) criteria. Head Neck..

[26] Russo D., Mariani P., Caponio V.C.A., Russo L.L., Fiorillo L., Zhurakivska K., Muzio L.L., Laino L., Troiano G. (2021). Development and Validation of Prognostic Models for Oral Squamous Cell Carcinoma: A Systematic Review and Appraisal of the Literature. Cancers.

[27] Bobdey S., Balasubramaniam G., Mishra P. (2016). Nomogram prediction for survival of patients with oral cavity squamous cell carcinoma. Head Neck..

[28] Montero P.H., Yu C., Palmer F.L., Patel P.D., Ganly I., Shah J.P., Shaha A.R., Boyle J.O., Kraus D.H., Singh B. (2014). Nomograms for preoperative prediction of prognosis in patients with oral cavity squamous cell carcinoma. Cancer.

[29] Bobdey S., Mair M., Nair S., Nair D., Balasubramaniam G., Chaturvedi P. (2018). A Nomogram based prognostic score that is superior to conventional TNM staging in predicting outcome of surgically treated T4 buccal mucosa cancer: Time to think beyond TNM. Oral Oncol..

[30] Li Y., Zhao Z., Liu X., Ju J., Chai J., Ni Q., Ma C., Gao T., Sun M. (2017). Nomograms to estimate long-term overall survival and tongue cancer-specific survival of patients with tongue squamous cell carcinoma. Cancer Med..

[31] Sun W., Cheng M., Zhuang S., Chen H., Yang S., Qiu Z. (2019). Nomograms to predict survival of stage IV tongue squamous cell carcinoma after surgery. Medicine.

[32] Chang B., He W., Ouyang H., Peng J., Shen L., Wang A., Wu P. (2018). A Prognostic Nomogram Incorporating Depth of Tumor Invasion to Predict Long-term Overall Survival for Tongue Squamous Cell Carcinoma With R0 Resection. J. Cancer.

